# Pathological clavicular fracture as first presentation of renal cell carcinoma: a case report and literature review

**DOI:** 10.7497/j.issn.2095-3941.2015.0033

**Published:** 2015-12

**Authors:** Yan Kong, Jin Wang, Huan Li, Peng Guo, Jian-Fa Xu, He-Lin Feng

**Affiliations:** ^1^Department of Medical Oncology, the Fourth Hospital of Hebei Medical University, Shijiazhuang 050011, China; ^2^Department of Orthopedics, the Fourth Hospital of Hebei Medical University, Shijiazhuang 050011, China; ^3^Postgraduate School, Tianjin Medical University, Tianjin 300070, China

**Keywords:** Clavicle, metastasis, renal cell carcinoma (RCC), pathological fracture

## Abstract

Renal cell carcinoma (RCC) accounts for approximately 3% of all cancer cases. RCCs usually metastasize to the lungs, bones, liver, or brain. Only <1% of patients with bone metastases manifested clavicular RCC metastases. Thus, clavicular metastasis as the initial presentation of RCC is extremely rare. We report a patient with RCC metastasis to the left clavicle, which was first presented with pain caused by a pathological fracture. Magnetic resonance image revealed a renal tumor, and technetium-99m–methylene diphosphonate bone scintigraphy showed multiple osseous metastases. The patient eventually underwent surgery to remove the lateral end of the left clavicle and right kidney. Histopathology revealed renal tumor and clear cell carcinoma in the clavicle. Finally, we review 17 cases of clavicular metastases originating from different malignancies.

## Introduction

Bone metastasis of renal cell carcinoma (RCC) is not rare but usually located in the spine, the pelvic bones, or the ribs^1^. A solitary bone metastasis and presented with a pathological fracture in the clavicle is extremely rare, with only a few references in the literature. We report one case of RCC presenting with a solitary clavicular metastasis and review the related literatures, so as to add to knowledge about clavicular metastasis.

## Case report

**Figure 1:**
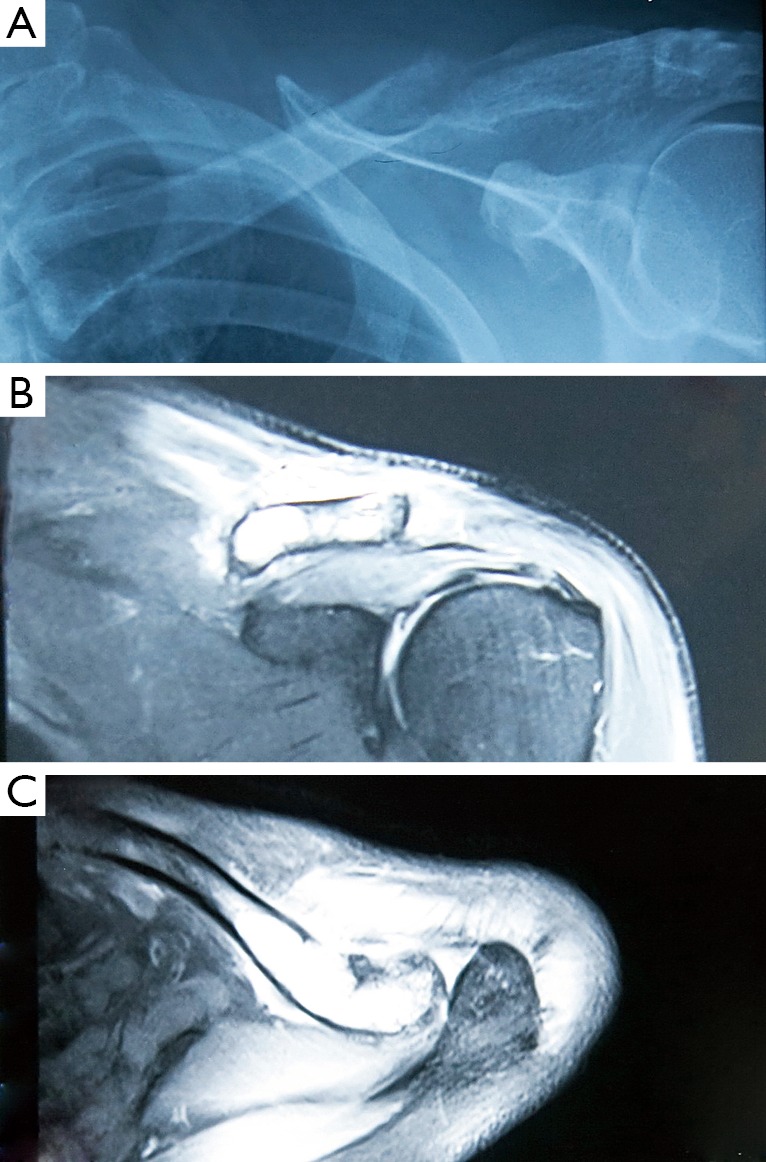
X-ray and MRI results of a 64-year-old patient. (A) X-ray result displayed an osteolytic lesion in the lateral third of left clavicle. (B,C) T2-weight MRI to the left clavicle revealed high-intensity lesions.

**Figure 2:**
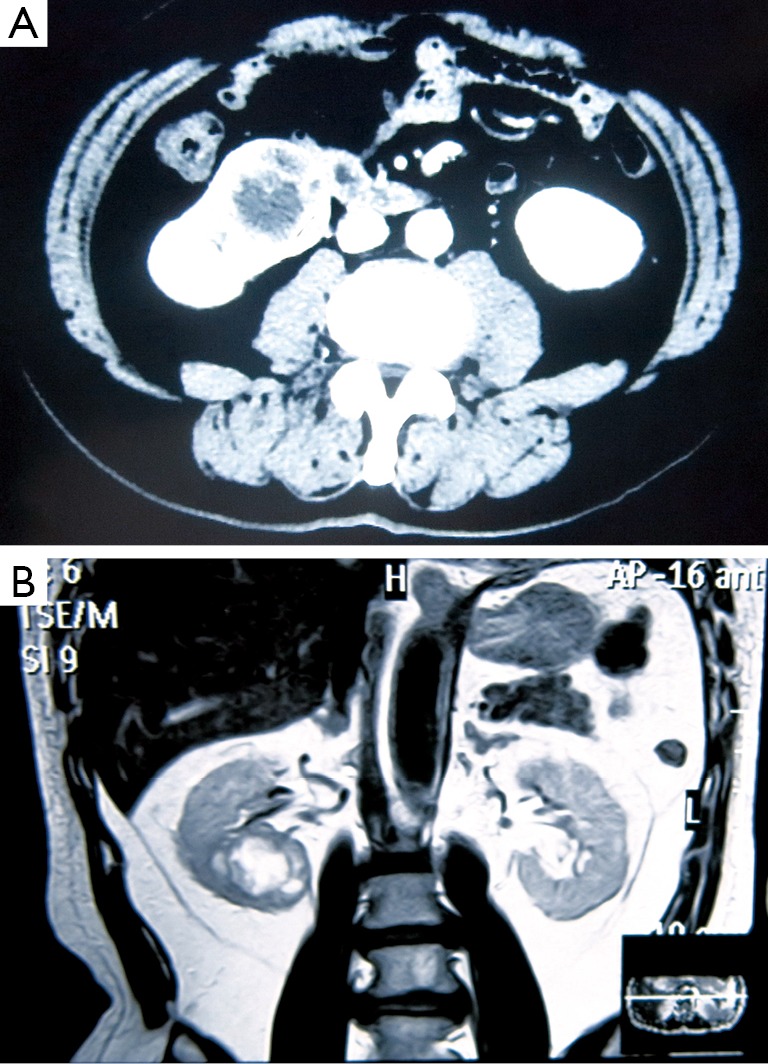
A tumor in the right renal cortex, spreading to the renal capsule. (A) CT. (B) MRI.

**Figure 3:**
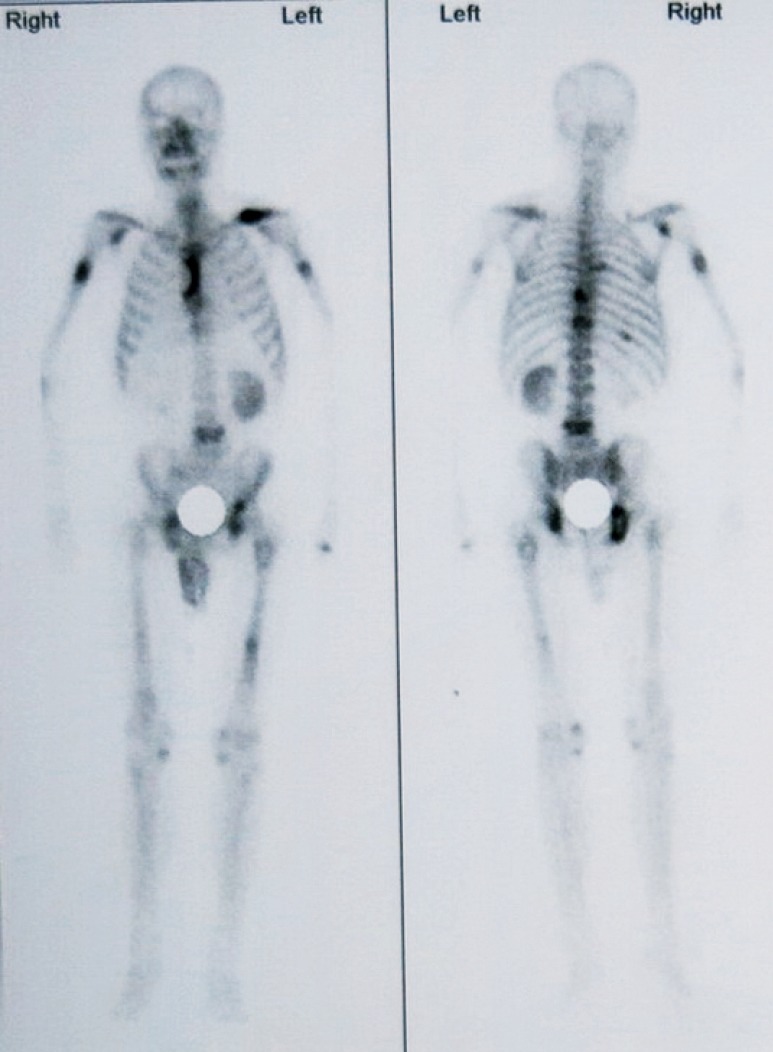
Technetium-99m–methylene diphosphonate bone scintigraphy revealed multiple radioactive foci.

A 64-year-old man was admitted to the Fourth Hospital of Hebei Medical University in January 2014, due to constant pain in the left shoulder after a mild trauma. X-ray and magnetic resonance imaging (MRI) results revealed a pathological clavicular fracture (**Figure 1**) because no specific, high-energy trauma in the clavicular region was reported and the lesion was osteolytic. Contrast-enhanced computed tomography (CT) and MRI images indicated a tumor, measuring approximately 5 cm × 5 cm, in the right renal cortex. The tumor had spread to the renal capsule (**Figure 2**). Technetium-99m–methylene diphosphonate bone scintigraphy revealed multiple radioactive foci, indicating multiple osseous metastases in addition to the clavicular metastasis (**Figure 3**).

**Figure 4:**
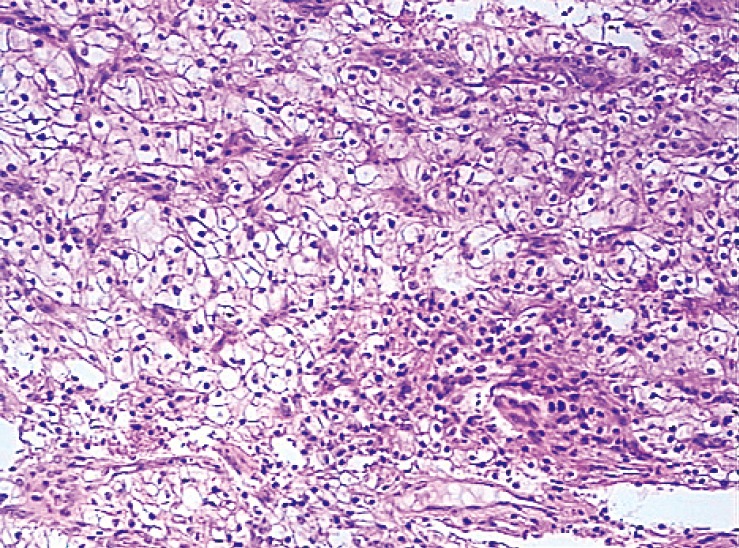
Histopathology of clavicle revealed clear cell carcinoma (H&E staining, 200×).

The patient underwent surgery to remove the lateral end of the left clavicle. Histopathology of the clavicle revealed clear cell renal carcinoma (**Figure 4**). The patient then underwent right nephrectomy, and histopathology showed RCC. Thus, clavicular RCC metastasis was confirmed. The patient was referred to an oncologist and was prescribed sorafenib tosylate and zoledronic acid. The patient was alive and showed no symptoms during a 6-month follow-up period.

## Discussion

RCC represents approximately 3% of all cancer cases. Classic symptom triad comprising hematuria, abdominal mass, and flank pain is found in only 10% of patients. However, RCCs commonly metastasize widely before any symptom is observed^1^. RCCs typically metastasize to the lungs, bones, liver, or brain, but clavicular involvement is reported in only <1% of all bone metastases^1^^-^^3^. Clavicular metastases are seldom reported. A total of 17 cases of clavicular metastases between 1998 and 2014 are reviewed in this study (**Table 1**)^4^^-^^8^^,^^10^^-^^18^. Despite four RCC cases^5^^,^^15^^,^^19^ which account for 25% of all documented cases, other malignancies rarely metastasize to the clavicle. Kim *et al.*^20^ studied 4,953 patients with hepatocellular carcinoma, of whom only 37 presented bone metastases and only one patient manifested clavicular metastasis. Osseous metastases from primary gastrointestinal cancers are also uncommon, ranging from 5.6% to 7.9%, and they usually occur in the vertebrae, pelvis, sacrum, skull, femur, and humerus^21^. A single case of malignant epithelioid schwannoma from the trigeminal nerve with clavicular metastasis has been documented, whereas metastases of intracranial gliomas to bones other than the clavicle are more frequent^13^. Although primary uveal and adnexal cancers rarely metastasize to the clavicle^4^^,^^13^, prostate and thyroid cancers commonly metastasize to this area^5^^,^^6^^,^^12^.

**Table 1:** Review of the previously reported cases of clavicular metastasis

Age of diagnosis (years)	FS	Sex	Side/location	Primary sites (histology)	Solitary or multiple	Ref.
48	N	Female	L/medial	RCC	Multiple	^4^
58	Y	Male	R/medial, lateral	RCC	Solitary	^5^
62	Y	Male	R/middle	RCC	Solitary	^5^
34	N	Male	R/medial	Uveal (melanoma)	Solitary	^6^
41	N	Female	L/medial	Thyroid (follicular)	Solitary	^7^
48	N	Male	R/-	Tonsil (SCC)	Multiple	^8^
67	Y	Male	R/medial	HCC (epithelial)	Solitary	^9^
68	N	Male	R/medial	Colon (AD)	Multiple	^10^
67	N	Male	L/medial	Cecum	Multiple	^11^
55	N	Female	-	Unknown (AD)	Multiple	^12^
67	N	Man	L/medial	Larynx (SCC)	Multiple	^13^
73	N	Female	L/medial	Adnexa (microcystic)	Multiple	^14^
20	N	Male	R/medial	Trigeminal (epithelioid schwannoma)	Multiple	^15^
67	N	Male	L/entire	Prostate	Solitary	^16^
52	N	Male	R/lateral	Adrenal (pheochromocytoma)	Solitary	^17^
40	N	Female	R/medial	Thyroid	Multiple	^18^
71	Y	Male	-	RCC	Multiple	^19^

FS, first presentation; RCC, renal cell carcinoma; SCC, squamous cell carcinoma; HCC, hepatocellular carcinoma; AD, adenocarcinoma.

Clavicular fractures represent 5% to 10% of all fractures^1^. Medial clavicular fractures are rare and are normally caused by high-energy traumas, which are frequently due to pathological causes^22^. Of the 17 cases, 11 had medial clavicular metastases^4^^-^^7^^,^^9^^-^^11^^,^^13^^-^^15^^,^^18^ and two of them revealed clavicular fracture^5^^,^^9^. Clavicular fracture was presented as the first symptom in four cases^5^^,^^9^^,^^19^. Swanson *et al*.^23^ reported that symptoms caused by bone metastases led to subsequent diagnosis of RCC in 121 of 252 (48%) patients, 37 of whom presented with pathological fractures. Our patient was admitted after a mild trauma that could not have caused clavicular fracture. This fracture should be evaluated carefully by physicians and requires routine investigation for an underlying pathological condition. Laboratory tests for measuring prostate-specific antigen, α-fetoprotein, blood calcium, and alkaline phosphatase levels; imaging tests, including radiography, CT, MRI, bone scan, and positron emission tomography; and fine-needle aspiration are essential for the early detection of primary tumors.

In conclusion, clavicular symptoms may be manifested before the diagnosis of primary tumors, such as RCC. Clavicular fracture may be the first symptom of tumor metastases to bones. Distinguishing pathological fractures from clavicular fractures due to other causes may help diagnose the primary tumors and necessitate whole-body scanning at an early stage.
